# Larval Diet Abundance Influences Size and Composition of the Midgut Microbiota of *Aedes aegypti* Mosquitoes

**DOI:** 10.3389/fmicb.2021.645362

**Published:** 2021-06-18

**Authors:** Hannah J. MacLeod, George Dimopoulos, Sarah M. Short

**Affiliations:** W. Harry Feinstone Department of Molecular Microbiology and Immunology, Bloomberg School of Public Health, Johns Hopkins University, Baltimore, MD, United States

**Keywords:** *Aedes aegypti*, microbiota, diet, nutrition, microbe, mosquito

## Abstract

The midgut microbiota of the yellow fever mosquito *Aedes aegypti* impacts pathogen susceptibility and transmission by this important vector species. However, factors influencing the composition and size of the microbiome in mosquitoes are poorly understood. We investigated the impact of larval diet abundance during development on the composition and size of the larval and adult microbiota by rearing *Aedes aegypti* under four larval food regimens, ranging from nutrient deprivation to nutrient excess. We assessed the persistent impacts of larval diet availability on the microbiota of the larval breeding water, larval mosquitoes, and adult mosquitoes under sugar and blood fed conditions using qPCR and high-throughput 16S amplicon sequencing to determine bacterial load and microbiota composition. Bacterial loads in breeding water increased with increasing larval diet. Larvae reared with the lowest diet abundance had significantly fewer bacteria than larvae from two higher diet treatments, but not from the highest diet abundance. Adults from the lowest diet abundance treatment had significantly fewer bacteria in their midguts compared to all higher diet abundance treatments. Larval diet amount also had a significant impact on microbiota composition, primarily within larval breeding water and larvae. Increasing diet correlated with increased relative levels of *Enterobacteriaceae* and *Flavobacteriaceae* and decreased relative levels of *Sphingomonadaceae*. Multiple individual OTUs were significantly impacted by diet including one mapping to the genus *Cedecea*, which increased with higher diet amounts. This was consistent across all sample types, including sugar fed and blood fed adults. Taken together, these data suggest that availability of diet during development can cause lasting shifts in the size and composition of the microbiota in the disease vector *Aedes aegypti*.

## Introduction

Mosquito-borne arboviruses like dengue virus, yellow fever virus and Zika virus are an ongoing public health concern, causing hundreds of millions of infections each year and tens of thousands of deaths (WHO, 2020). The vast majority of arboviral diseases have no vaccine and treatment is limited to supportive care. Additionally, novel arboviruses continue to emerge, presenting a “moving target” for public health intervention. One commonality for all these pathogens is transmission by vector arthropods such as the yellow fever mosquito *Aedes aegypti*, and disease prevention efforts focus on reducing transmission through vector population reduction or replacement strategies (Wilson et al., 2020).

The mosquito midgut microbiota has been shown to influence factors relevant to vector-borne disease transmission (Minard et al., 2013; van Tol and Dimopoulos, 2016; Guégan et al., 2018) including larval development (Coon et al., 2014, 2017; Correa et al., 2018), susceptibility to arboviral infection (Xi et al., 2008; Apte-Deshpande et al., 2012, 2014; Ramirez et al., 2012, 2014; Wu et al., 2019; Möhlmann et al., 2020), blood digestion (Gaio et al., 2011), egg production (Gaio et al., 2011; Gendrin et al., 2015), and longevity (Bahia et al., 2014; Ramirez et al., 2014; Gendrin et al., 2015, 2016). These observations have led to growing interest in manipulation of the midgut microbiota for vector control and/or prevention of disease transmission. Progress in this regard would be greatly facilitated by a more complete understanding of the factors governing microbiota size and composition in the mosquito.

Previous work suggests that the environment is an important determinant of mosquito microbiota formation. For example, bacterial populations in breeding sites have been shown to correlate with midgut microbiota composition in larvae (Coon et al., 2014, 2016; Gimonneau et al., 2014), and larvae have been shown to ingest bacteria regularly during development (Coon et al., 2017). These data support the hypothesis that the larval microbiota is orally acquired and reflective of the bacterial community in the larval breeding water. The adult microbiota is also influenced by breeding site (Gimonneau et al., 2014; Buck et al., 2016), and bacteria found in larvae are commonly also found in adults (Coon et al., 2014; Guégan et al., 2018), suggesting adult microbiota is influenced at least in part by the microbial community in the larval breeding water.

Environmental factors influencing the microbial communities in larval breeding water could, therefore, have a long-lasting impact on the mosquito microbiota. It remains unclear, however, what aspects of the larval breeding habitat influence microbiota formation in larvae or adults. At least two studies have assessed the impact of larval diet on microbiota formation in mosquitoes. One found that the type of food (e.g., fish food flakes vs. fish food pellets) available to larvae had a significant and lasting impact on the amount of *Enterobacteriaceae* and *Flavobacteriaceae* in larval and adult *Anopheles gambiae* and a correlated impact on susceptibility to infection by *Plasmodium* parasites (Linenberg et al., 2016). Another found that varying the amount of diet provided to *Culex nigripalpus* larvae in outdoor mesocosms had no significant effect on microbiota composition in larvae or adults, though they did identify indicator species that corresponded with high and low organic matter treatments (Duguma et al., 2017). To our knowledge, there is no comparable information in *A. aegypti*, a critical vector of human arboviruses.

We assessed how the amount of diet available during development influences both the composition and size of the microbiota during different life history stages for *A. aegypti* mosquitoes. To test this, we reared *A. aegypti* mosquitoes with access to varying amounts of a complex larval diet and assessed the total bacterial load as well as the composition of the microbial community in breeding water, larvae, and the midguts of sugar fed and blood fed adult females. We found that lower diet abundance was generally predictive of a smaller bacterial community in larvae and that this effect persisted through adulthood and even after a blood meal. We also found that microbiota composition was significantly affected by larval diet availability. This was primarily observed in breeding water and larvae, though for some bacterial taxa the effects persisted into adulthood. Among all sample types, estimates of within-sample diversity (alpha diversity) were positively correlated with diet abundance. Additionally, diet was a significant predictor of diversity between samples (beta diversity), i.e., the microbiota composition of samples from the same feeding regimens were more similar to each other than to samples from different feeding regimens. These findings suggest that, for *A. aegypti*, the amount of food in breeding water can significantly influence the microbiota over multiple stages of development and into adulthood, when these mosquitoes are capable of transmitting pathogens to humans.

## Materials and Methods

### Experimental Design, Sample Collection, and Sample Processing

#### Mosquito Strain Maintenance

*Aedes aegypti* Singapore (Sing) mosquitoes were established from larvae collected in the field in Singapore in 2010 (Sim et al., 2013). For strain maintenance, we reared Sing strain larvae at 27°C and 80% residual humidity on a 14:10 light:dark photocycle. We reared larvae in reverse osmosis (RO) water with *ad libitum* access to larval food (liver powder, tropical fish flake food, and rabbit food pellets mixed in a 2:1:1 ratio and autoclaved) and provided adults *ad libitum* access to 0.22 μm filter-sterilized 10% sucrose.

#### Experimental Design and Replication Structure

For each full biological replicate of the experiment, we reared one tray of larvae per diet treatment and transferred pupae from each tray to a single cage (4 total trays/cages). We set up three full biological replicates for a total of 12 trays/cages (4_f__eeding regimens_ × 3_b__iological replicates_ = 12_t__rays/cages_). To rear mosquitoes for the experiment, we bleached eggs once with 3% bleach then rinsed 2X with RO water, and hatched them in a vacuum. Development of larvae from the lowest R1 feeding regimen is delayed by 1 day, so we hatched eggs for this treatment 1 day earlier than for the other treatments. Eggs for all treatments were laid by the same two generations (F24–F25) of Sing strain adults. For all treatments, we thinned larvae immediately after hatching to 200 larvae per tray and to each tray added 2L RO water. In addition, we added 500 μL of a 25% glycerol stock of breeding water collected from an *Aedes* mosquito breeding site (tire pile) in Baltimore, MD on September 23, 2016, to standardize the starting breeding water microbiota across diet regimens and make it more reflective of a natural *Aedes* container breeding site. Since collection, the glycerol stock has been continuously stored at −80°C and handled under sterile conditions. Larval food, prepared as described above, was replenished daily under the food regimens shown in Table 1, which were based on those described in Yeap et al. (2011) and ranged from nutritional deprivation to excess. When the larvae were 4th instars, we collected *n* = 2 water samples from each tray and *n* = 3 larval samples (5 larvae/sample) from each tray. We then transferred pupae from each tray to a separate cage and allowed adults to eclose. Adults were maintained on 10% sucrose until dissection. From each cage, we collected *n* = 3 pooled midgut samples from sugar fed adult females (8 midguts/sample) and *n* = 1–3 pooled midgut samples from blood fed adult females (average six midguts/sample). Our design therefore included biological replicates (i.e., separate trays/cages) as well as technical replicates (i.e., replicate samples taken from the same tray or cage). A listing of all samples used in the experiment can be found in Supplementary Table 1. All samples were used for qPCR analysis. Samples from R1, R2, and R4 were used for 16S amplicon sequencing. All technical replicate pools were sequenced from R1 and R2 to verify that microbiota composition did not vary by technical replicate. Final 16S profiling analysis was performed using the first technical replicate from all treatments to ensure the same number of samples were used from all treatment groups.

**TABLE 1:** Amount of larval food per larva per day for four feeding regimens.

	Days 0–1 post hatching	Day 2 post hatching – pupation
Feeding regimen	Diet conc. (mg/larva/day)	Diet conc. (mg/larva/day)
R1	0.125	0.25
R2	0.375	0.75
R3	0.5	1
R4	1	2

#### Water and Larval Collection and Sample Preparation

When larvae were 4th instars, we collected two samples of 5 mL of larval water from each tray in a conical bottom tube. We then centrifuged all samples at 5,000 × g for 20 min at 4°C, removed the supernatant, and froze the pellet at −20°C for storage. On the same day, we removed 15 4th instar larvae from each tray and transferred them by treatment to separate wells of a 6-well cell culture plate. We then immobilized larvae on a cold block, surface sterilized them with 70% EtOH, and washed them twice with sterile 1X PBS. We transferred three pools of five larvae from each group to 200 μl lysis solution from the Zymobiomics DNA extraction kit (Zymo Research, Irvine, CA, United States), homogenized with sterile pestles, and stored the samples at −80°C. We also collected contamination control buffer blanks (lysis buffer handled identically to an experimental sample but without tissue added) for each biological replicate. We collected water and larval samples before supplementing the larval breeding water with food for the day.

#### Adult Blood Feeding, Sample Collection, and Preparation

For blood feeding, we starved females overnight and then provided them a blood meal consisting of 45% human red blood cells and 55% heat-inactivated human serum. The blood meal was also supplemented with 1% 100 mM ATP. Sugar fed females were also starved and then provided 10% sucrose meals the following morning. Adult females were dissected at 4–6 days post eclosion and 24 h after blood feeding. Sugar fed females were dissected in parallel with blood fed females. We first removed the right wing from eight sugar fed females from each larval diet/replicate combination for a total of 24 wings per larval diet treatment. We then returned females to their respective groups and externally sterilized all adult females with 70% EtOH for a minimum of 30 s, then washed them twice with filter-sterilized 1X PBS. We dissected midguts from each mosquito on glass slides (sterilized with 70% EtOH) in sterile 1X PBS. We cleaned forceps with 70% EtOH between pools of mosquitoes and used clean 1X PBS for each dissection pool. We transferred dissected midguts to 200 μL lysis solution from the ZymoBIOMICS DNA Miniprep Kit (Zymo Research, Irvine, CA, United States) in microcentrifuge tubes on ice. We also collected contamination control buffer blanks (lysis buffer handled identically to an experimental sample but without tissue added) for each biological replicate. Samples were then frozen at −80°C until DNA extraction. Our goal was to dissect three pools of eight females from each diet/replicate/adult feeding status combination, which we were able to achieve for nearly all samples from the sugar fed treatment. For blood fed females, however, we were unable to obtain three pools of eight in many instances because not enough mosquitoes took a blood meal. Those differences are documented in Supplementary Table 1.

#### DNA Extraction

DNA was extracted using the ZymoBiomics DNA Miniprep Kit according to the manufacturer’s instructions, with the following adjustments: all samples were homogenized manually using sterile pestles before the bead beating step. Pestles were treated with DNA erase (Sigma) and rinsed with sterile water prior to use. DNA was eluted in 100 μL filter-sterilized water heated to 60°C.

#### Wing Measurement

We measured wing length as a proxy for body size to compare size of adults between feeding regimens (Christophers, 1960; Bock and Milby, 1983; Van Handel and Day, 1989). We mounted wings on microscope slides using double sided tape and used ImageJ to measure the distance between the alular notch and the distal end of the right mosquito wing (i.e., the termination of the R3 wing vein), according to Bock and Milby (1983).

#### qPCR

To quantify the bacterial load in our samples, we performed qPCR targeting the bacterial 16S rRNA gene. For all samples, in each well we combined 7.5 μL SYBR master mix (Applied Biosystems), 0.35 μL of each primer (primer starting concentrations were all 10 μM), 1 or 5 μL template (as described below), and MilliQ water to a final volume of 15 μL. qPCR conditions were as follows: 95°C for 10 min, (95°C for 15 s then 60°C for 1 min) × 40 cycles. A melt curve was performed after all reactions to verify single product amplification. Primers used for qPCR can be found in Supplementary Table 2.

For water samples, we diluted gDNA 1:100 and used 1 μL of gDNA as template, and all water samples were run in quadruplicate. This was necessary for water samples because in an initial qPCR run with duplicated wells, multiple samples had more than 1 CT difference between duplicate wells and had to be discarded. We then repeated the qPCR run to obtain either two or four high quality technical replicates for each sample. 16S copy number per microliter was determined using a standard curve generated from gel-purified PCR product of the *E. coli* 16S gene. We quantified the amount of DNA in ng/μL in our PCR product using a NanoDrop 2000 (Thermo Scientific) then, using the length of the PCR product (466 bp, Nadkarni et al., 2002) and assuming an average weight per base pair of 660 Da, we determined the copies per μL in our undiluted PCR product and subsequent dilutions. We then compared the CT values of our water samples to this standard curve to determine 16S copy number per microliter for each water sample. Technical replicates were averaged for each sample and the average copy number concentrations (copies per μL) were then used for data analysis (see below).

For tissue samples (larvae, adult sugar fed midguts, adult blood fed midguts), we diluted gDNA 1:50 then used 5 μL of gDNA as template. We performed qPCR targeting both the bacterial 16S rRNA gene and the mosquito S7 gene (AAEL009496), and all reactions were run in duplicate. See Supplementary Table 2 for primer sequences. All technical replicates were averaged, and S7 CT values were subtracted from 16S CT values for each sample to obtain delta CT values. Delta CT values were used for data analysis (see below). Inverse delta CT values were used in plots.

#### 16S Amplicon Sequencing and Data Processing

The concentration of DNA samples was determined using a NanoDrop 2000 (Thermo Fisher Scientific), and used to dilute samples to 1 ng/μl; 5 ng of DNA was used as template in a PCR to amplify the V4 region of the bacterial 16S rRNA gene. Primers were 515F and 806R with Illumina adapters and eight basepair dual indices (Kozich et al., 2013), and all reactions were performed in triplicate using GoTaq (Promega) and including 10 μg BSA (New England Biolabs). We also added 0.1 femtomole 515F and 806R without adapters or barcodes. This was done to overcome initial primer binding inhibition. PCR reaction conditions were as follows: 95°C for 2 min, then 30 cycles of 30 s at 95.0°C, 60 s at 50.0°C, and 60 s at 72.0°C, followed by final extension as 72.0°C for 10 min. Four samples (samples 10, 21, 22, and 23, Supplementary Table 1) did not amplify under these conditions, so an additional five cycles were performed for these samples. PCR products were quantified and visualized using the QIAxcel DNA Fast Analysis (Qiagen). Negative buffer controls (generated by performing a DNA extraction on lysis solution handled identically to tissue samples during larval and adult sample collection) failed to amplify but were still included in the sequencing reaction to account for any potential contamination. PCR products were normalized based on the concentration of DNA from 350 to 420 bp then pooled using the QIAgility liquid handling robot. The pooled PCR products were cleaned using the Mag-Bind RxnPure Plus (Omega Bio-tek) according to the manufacturer’s protocol. The cleaned pool was sequenced on a MiSeq system using the MiSeq Reagent Kit V2 (Illumina, Inc.).

Sequences were demultiplexed using onboard bcl2fastq. Demultiplexed sequences were processed in Mothur v. 1.39.4 following the MiSeq SOP (Kozich et al., 2013), and exact commands can be found here: https://github.com/krmaas/bioinformatics/blob/master/mothur.batch. Merged sequences that had any ambiguities or did not meet length expectations were removed. Sequences were aligned to the Silva nr_v119 alignment (Quast et al., 2013). Taxonomic identification of OTUs was done using the RDP Bayesian classifier (Wang et al., 2007) against the Silva nr_v119 taxonomy database. OTUs were determined using the opti clustering method with a distance cutoff of 0.03 (97% similarity).

### Data Analysis

#### Wing Length Data Analysis

To assess the effect of diet regimen on wing length, we fit a linear mixed-effect model using lme in the package “nlme” in R (Pinheiro et al., 2019). The response variable was wing length in millimeters and we used diet regimen as a fixed effect and biological replicate as a random effect. We measured wings from eight individuals per diet regimen per biological replicate, for a total of 24 measurements per diet regimen. After fitting the overall model, we performed an ANOVA querying the effect of diet regimen followed by a Tukey’s test using the glht function in the package “multcomp” in R (Hothorn et al., 2008) to compare wing lengths between each diet regimen. Models and outputs can be found in Supplementary File 1. Raw data can be found in Supplementary File 2.

#### qPCR Data Analysis

To assess the effect of diet regimen on bacterial 16S copy number, we fit linear mixed-effect nested models using lme in the package “nlme” in R (Pinheiro et al., 2019). Separate models were fitted to breeding water data, larval data, and adult data. Both sugar fed and blood fed adults were analyzed together since they were sampled at the same time. The full model included average 16S copy number as the response variable, larval diet as a fixed effect and biological replicate and technical replicate (pool) as random effects. For adult data, we also included adult feeding status as a fixed effect. Biological replicate is defined as larval tray/cage per diet level (3 trays/cages = 3 biological replicates). Technical replicates are the individual pools taken per tray/cage. Technical replicate was nested within biological replicate in all analyses. After fitting the overall model, we performed an ANOVA to determine the overall significance of fixed effects, and then performed a Tukey’s test using the glht function in the package “multcomp” in R (Hothorn et al., 2008) to assess pairwise differences between diet regimens. Models and outputs can be found in Supplementary File 1. Raw data can be found in Supplementary File 2.

#### Sequencing Data Analysis

Sequencing data analysis was primarily performed using the phyloseq and vegan packages in R (McMurdie and Holmes, 2013; Oksanen et al., 2019). Sequences were filtered to remove any taxa that did not map to bacteria and all taxa that contained fewer than 0.005% of all reads in the dataset (Bokulich et al., 2013). To assess the potential for contamination, Bray Curtis dissimilarity values were calculated and a non-metric multidimensional scaling (NMDS) analysis performed to compare the buffer blanks to all experimental samples. To determine whether sequencing was repeatable across technical replicates, a PERMANOVA was performed on all samples assessing the effect of technical replicate nested within biological replicate. No significant effect of technical replicate was detected, suggesting microbiota composition is consistent across technical replicates. To standardize sample number across all treatments, for all downstream analyses only the first technical replicate was used. Therefore, the final sequencing dataset included three diets (R1, R2, and R4) and four sample types (breeding water, larvae, adult sugar fed, adult blood fed), and for all combinations, three biological replicates were analyzed. Each biological replicate consisted of a pool of 5–8 individuals; samples used in final analysis and number of individuals per pool can be found in Supplementary Table 1. Effect of diet and sample type on relative abundance of individual OTUs was performed using Analysis of Composition of Microbiomes (ANCOM) (Mandal et al., 2015). This analysis method allows for the inclusion of random effects (biological replicate in our case) and corrects for multiple comparisons to control for the fact that we are testing the effect of diet on each OTU in our dataset. This was first performed on all samples combined and then, given the dramatic differences in composition between sample types, on breeding water/larval samples alone. ANCOM would not successfully run on adult samples, likely due to the highly unbalanced nature of the adult sample dataset (most reads fall into very few OTUs). This persisted even after repeated attempts to trim the dataset to reduce low frequency and zero-count OTUs, suggesting it is an inherent problem in the structure of the dataset and not a filtering issue. The dataset was then scaled to standardize the number of reads in each sample using a method developed by Denef et al., 2016. This approach achieves the same result as rarefying the dataset. The scaled dataset was used for alpha and beta diversity analyses. Alpha diversity indices were generated using the estimate_richness command in phyloseq. Main effects of diet and treatment and an interaction between the factors was assessed for each index using an ANOVA. To assess beta diversity, Bray Curtis dissimilarity values were calculated and NMDS analysis performed using the ordinate function in phyloseq. PERMANOVA was performed using adonis in vegan. All R code, notes, and outputs for microbiota composition analysis can be found in Supplementary File 3.

## Results

### Mosquitoes Reared With Lower Abundance of Larval Diet Host a Smaller Microbiota Even After Accounting for Reduced Mosquito Body Size

**FIGURE 1:**
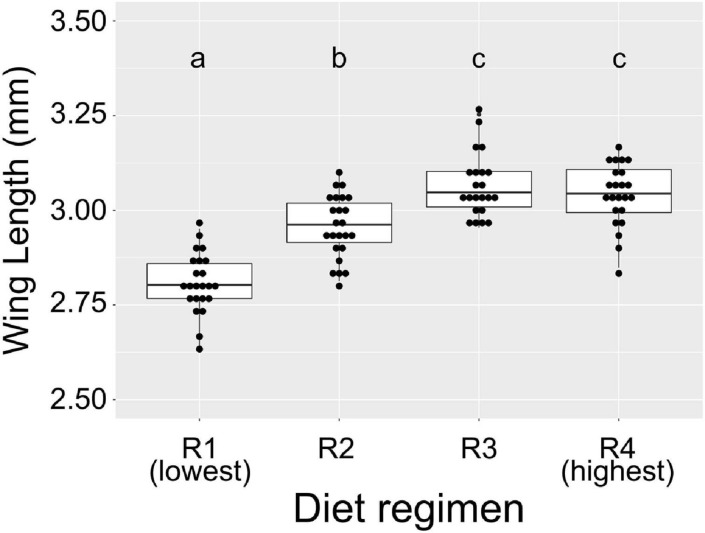
Diet abundance during larval development significantly affects adult wing size. We measured wing length (in mm) of adult female *A. aegypti* reared under different larval diet amounts. Amount of diet was lowest in R1 and increased through R4 (Table 1). Results are from eight individuals per biological replicate (*n* = 24 per treatment). Data were analyzed using a linear mixed model with diet regimen as a fixed effect and biological replicate as a random effect. ANOVA indicated a significant effect of diet on wing length (*F* = 46.11, *p* < 0.0001). Significant pairwise differences (*p* < 0.01) between treatments were assessed using Tukey’s test and are denoted by letters above each treatment.

First, it was important to assess whether larval diet abundance affects body size, because differences in body size among the diet treatments could influence total bacterial load as well. We measured wing length of adults from the alular notch to the distal end to estimate overall body size. We used a linear mixed-effects model to assess the impact of diet on overall wing size and, as expected (Yeap et al., 2011), found that diet significantly predicted wing size (*F* = 46.11, *p* < 0.0001). Pairwise comparisons using a Tukey’s test showed that adults reared with the lowest access to food (the “R1” group, Table 1) had significantly shorter wings than all other groups (Figure 1). Similarly, individuals with the next highest access to food (the “R2” group, Table 1) had significantly longer wings than R1 individuals but significantly shorter wings than individuals from R3 and R4 (Figure 1). Individuals reared under higher larval food abundance (R3 and R4) were not significantly different from each other. Significant differences in wing size range from a 2.7% increase (R2 vs. R4) to an 8.9% increase (R1 vs. R3) (Supplementary Table 3), which does reflect a potential difference in size of the midgut between treatments. To account for this issue, all 16S qPCR values were corrected for expression of S7, a housekeeping gene, which controls for potential differences in tissue amounts.

**FIGURE 2:**
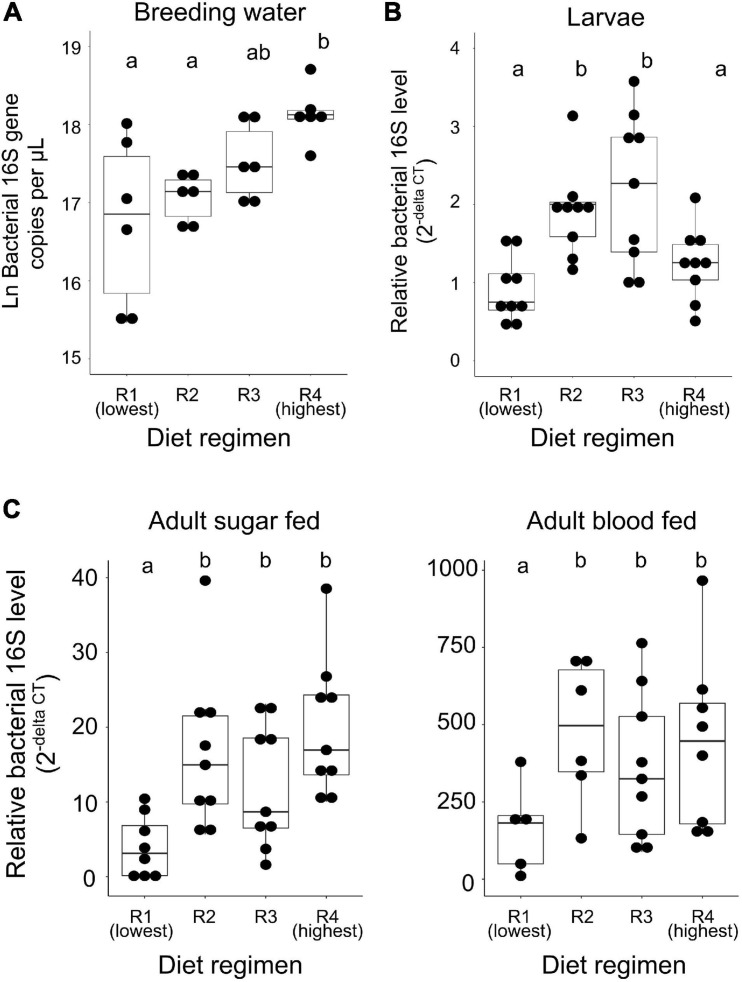
Microbial community size is restricted by low diet availability during larval development. We reared *A. aegypti* larvae with varying access to larval diet (R1 = lowest abundance, R4 = highest, Table 1) and used 16S qPCR to quantify bacterial load in breeding water, whole larvae, sugar fed adult midguts, and blood fed adult midguts. Data were analyzed using mixed effect nested linear models to determine the effect of diet regimen on 16S level. Results of pairwise comparisons are denoted by letters above each treatment. **(A)** Higher diet availability results in higher 16S levels in breeding water (*F*-value = 5.537, *p* = 0.0092). For each diet, *N* = 6 (two samples from each of three replicate breeding trays). **(B)** Food availability significantly impacts 16S levels in larvae (*F*-value = 16.734, *p* < 0.0001). For each diet, *N* = 9 (three samples from each of three replicate breeding trays). **(C)** Low food availability during larval development results in lower 16S levels in sugar fed and blood fed adult midguts (*F*-value = 15.934, *p* < 0.0001); 16S levels are also significantly predicted by blood feeding status (*F*-value = 168.036, *p* < 0.0001). For each diet/feeding status combination, *N* = 9 (three samples from each of three replicate cages) with the exceptions of R1/sugar fed, *N* = 8; R1/blood fed, *N* = 5; R2/blood fed, *N* = 6; R4/blood fed, *N* = 8.

Next, we quantified total bacterial load in breeding water, whole larvae, and adult mosquito midguts from all larval feeding regimens using qPCR targeting the bacterial 16S rRNA gene. We then used linear mixed effect models to determine the effect of feeding regimen on total bacterial load in each sample type. In larval breeding water, we found that diet was a significant predictor of bacterial 16S rRNA gene copy number (Figure 2A; *F*-value = 5.537, *p* = 0.0092). When comparing all diet regimens pairwise, we found that R4 samples (the highest diet amount) had significantly higher 16S copy number than samples reared in both R1 (*p* < 0.001) and R2 (*p* = 0.016) diet regimens. No other pairwise comparisons were significant. Among larvae, we found that larval diet was a significant predictor of bacterial 16S relative abundance (Figure 2B; *F*-value = 16.734, *p* < 0.0001). In pairwise comparisons, we found that the bacterial load of whole larvae did not differ significantly between individuals from the R1 and R4 treatments (*p* = 0.092), but was significantly higher in individuals from the R2 and R3 treatments compared to those from both the R1 and R4 treatments (Figure 2B; R1 vs. R2, *p* < 0.0001; R1 vs. R3, *p* < 0.0001; R4 vs. R2, *p* = 0.004; R4 vs. R3, *p* < 0.0001). Among adults, we found that both larval diet (*F*-value = 15.934, *p* < 0.0001) and blood feeding status (*F*-value = 168.036, *p* < 0.0001) significantly predicted relative 16S rRNA levels but that there was no interaction between the two factors, suggesting that the effect of diet is consistent regardless of blood feeding status (Figure 2C). We therefore removed the interaction from the model and performed pairwise comparisons between diet levels regardless of blood feeding status. We found that individuals from the R1 treatment had significantly lower midgut bacterial loads than individuals from all other treatments and that the other treatments were not significantly different from one another (Figure 2C; R1 vs. R2, *p* < 0.0001; R1 vs. R3, *p* < 0.0001; R1 vs. R4, *p* < 0.0001; R2 vs. R3, *p* = 0.770; R2 vs. R4, *p* = 0.995; R3 vs. R4, *p* = 0.589).

### Larval Diet Abundance Induces Significant Shifts in Bacterial Community Composition

In order to assess the effects of larval diet abundance on microbial community composition, we performed high throughput 16S amplicon sequencing on breeding water, whole larvae, sugar fed adult midguts, and blood fed adult midguts from feeding regimens R1, R2, and R4. Average number of sequences obtained per experimental sample after all filtering steps was 44,180.2 and samples ranged from 6,647 to 83,125 total reads (Supplementary File 3). The blank samples we collected (which consisted of lysis solution handled identically to larval and adult tissue samples during collection) failed to amplify in PCR and yielded very few sequences (mean = 144.5; range: 66–361 total reads) which suggests that contaminants are likely to be, on average, approximately 0.3% of the reads in our experimental samples. We performed a NMDS analysis to evaluate similarity between our experimental samples and blanks, which showed that blanks cluster together and separately from experimental samples (Supplementary Figure 1). We therefore proceeded with analysis despite the presence of this minor contamination.

**FIGURE 3:**
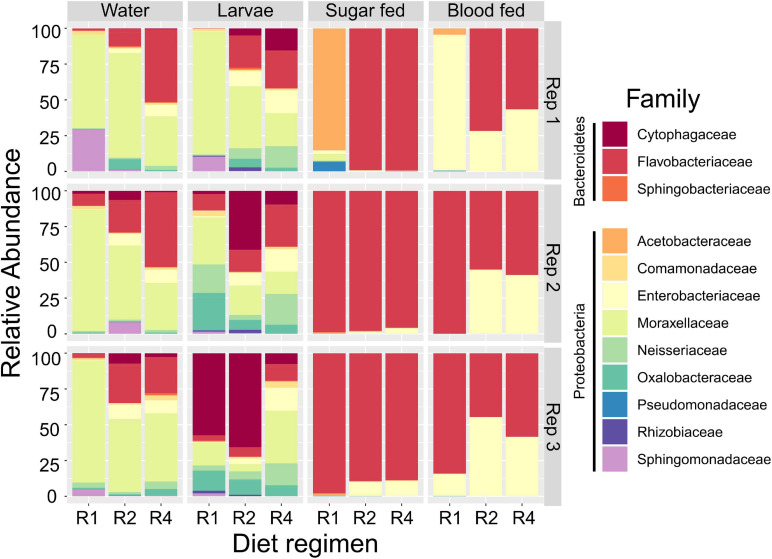
Larval diet abundance impacts microbiota composition. Percent composition of bacterial families in larval breeding water, larvae, adult sugar fed midguts, and adult blood fed midguts from three feeding regimens (R1, R2, and R4, Table 1) and three biological replicates. Taxa containing fewer than 0.1% of all reads in the entire dataset were excluded.

Our initial OTU assignment at 97% similarity resulted in 76 OTUs but 99.9% of the total reads fell into 19 OTUs, indicating simple microbial communities among all experimental samples (Figure 3). Communities were composed primarily of the phyla Proteobacteria (44.3% of all reads) and Bacteriodetes (55.5% of all reads). Families most commonly found among our samples were *Flavobacteriaceae* (50.2% of all reads), *Moraxellaceae* (20.8% of all reads), *Enterobacteriaceae* (15.8% of all reads), *Cytophagaceae* (5.0% of all reads), *Oxalobacteraceae* (1.8% of all reads), Neisseriaceae (1.6% of all reads), *Sphingomonadaceae* (1.6% of all reads), and *Acetobacteraceae* (1.1% of all reads) (Figure 3). All other families accounted for less than 1% of all reads. Initial examination of relative abundance data at the family level showed that bacterial communities were more diverse in water and larval samples than in adults, which primarily consisted of only two families (*Flavobacteriaceae* and *Enterobacteriaceae*). Taxa from the family *Enterobacteriaceae* increased in relative abundance with increased larval diet amount, as did taxa from *Flavobacteriaceae*, but only in breeding water and larvae (Figure 3).

**FIGURE 4:**
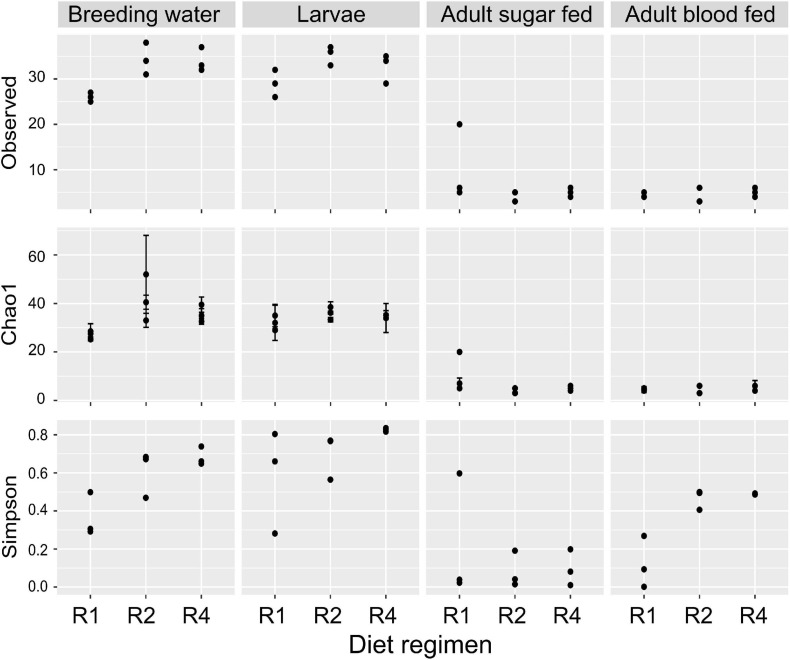
Diet during larval breeding impacts alpha diversity. We measured alpha diversity across three larval diets (R1, R2, and R4) and four sample types (breeding water, larvae, adult sugar fed, adult blood fed). We performed a two-factor ANOVA including diet, sample type, and diet/sample type interaction as factors. For observed OTUs, the interaction of diet and sample type was not significant (*F* = 2.193; *p* = 0.08), nor was the main effect of diet (*F* = 0.296; *p* = 0.7459). Sample type, however, was significant (*F* = 106.446; *p* = 4.38 × 10^–16^). For Chao1 values, the interaction of diet and sample type was significant (*F* = 3.847, *p* = 0.008). For Simpson’s index (1-D), the interaction of diet and sample type was not significant (*F* = 2.174; *p* = 0.08) but there was a significant effect of diet regimen (*F* = 4.745, *p* = 0.016) and sample type (*F* = 20.193; *p* = 2.35 × 10^–7^).

We assessed whether alpha diversity (i.e., microbial community diversity within samples) differed between diet regimens and sample types, using measures of richness (observed OTUs and Chao1 index) and Simpson’s index (1-D), which accounts for richness and evenness. Observed richness did not significantly differ by diet regimen (*F* = 0.296; *p* = 0.746) but sample type was highly significant (*F* = 106.45; *p* = 4.38 × 10^–16^; Figure 4). The observed number of OTUs was highest in larval breeding water and larvae and substantially lower in sugar fed and blood fed adult midguts (Figure 4). For Chao1, we observed a significant interaction between diet regimen and sample type (*F* = 3.847; *p* = 0.008), indicating that the effect of diet regimen on estimated richness differed by sample type. Upon further inspection of the data, we found that Chao1 index values were higher in treatments with higher larval diet concentrations, but this effect was primarily limited to the breeding water (Figure 4). Diet regimen significantly predicted Simpson’s Index value (*F* = 4.74, *p* = 0.016) and sample type was highly significant (*F* = 20.19, *p* = 2.35 × 10^–7^); there was no significant interaction between diet regimen and sample type, suggesting any effect of diet regimen is consistent across sample types (Figure 4). Simpson’s index was lowest in samples from the R1 treatment and higher in R4 samples (Figure 4).

**FIGURE 5:**
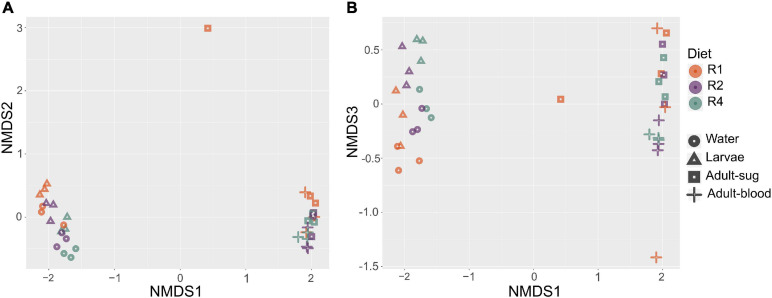
Non-metric multidimensional scaling analysis reveals clustering by sample type and larval diet regimen. We performed NMDS analysis using Bray-Curtis dissimilarity values. Plots show axes 1 and 2 **(A)** and axes 1 and 3 **(B)**. Color is used to indicate diet regimen (orange = R1, purple = R2, green = R4) and shape is used to indicate sample type (circle = breeding water, triangle = larvae, square = adult sugar fed midguts, cross = adult blood fed midguts). Three biological replicate samples are plotted for each diet regimen-sample type combination.

We also assessed beta diversity (i.e., differences in microbial community between samples) in our samples and the effects of diet and sample type on beta diversity. Using the subsampled dataset, we performed a NMDS analysis on all samples using Bray Curtis dissimilarity as the distance metric. This revealed clustering by sample type, with breeding water and larvae clustering together and sugar fed and blood fed adults clustering together (Figure 5A). This analysis also suggested clustering by diet, and this effect appeared to be mostly limited to breeding water and larval samples (Figure 5B). To test whether larval diet or sample type significantly predicted differences in microbiota composition between samples, we performed a PERMANOVA on Bray Curtis dissimilarity values to assess the effect of diet (R1, R2, or R4) and sample type (breeding water, larvae, sugar fed adult, blood fed adult). We found a significant overall effect of diet (*p* = 0.003) and sample type (*p* = 0.001) and the interaction between these factors was not significant (*p* = 0.856).

**FIGURE 6:**
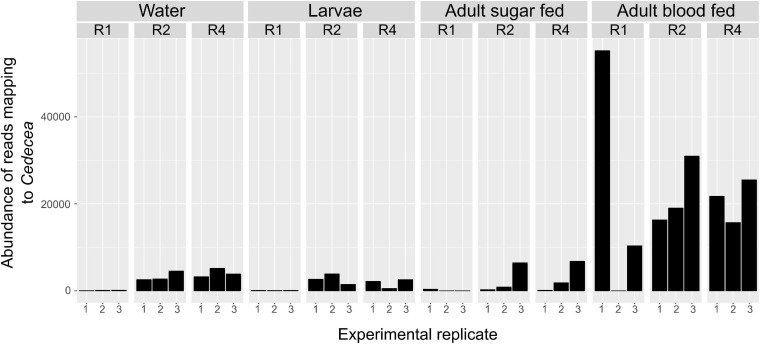
Abundance of *Cedecea* is significantly affected by larval diet amount in all sample types. Abundance of one OTU that mapped to the genus *Cedecea* was significantly affected by larval diet amount (W = 58, significant at 0.8 cutoff threshold). Abundance is plotted for all sample types, diet treatments, and biological replicates. Abundance is lowest in R1 in general and higher in R2 and R4 treatments. This is consistent across all sample types and replicates with the singular exception of the first replicate of adult blood fed samples.

Our beta diversity analysis indicated that overall microbial community composition varied significantly between diets. We therefore determined whether the prevalence of specific OTUs was significantly affected by diet using ANCOM. When we analyzed all sample types together (breeding water, larvae, sugar fed adults, and blood fed adults), we found that diet significantly affected the abundance of only one OTU (OTU0003, W = 58, significant at 0.8 cutoff threshold) which mapped to the family *Enterobacteriaceae* and the genus *Cedcea* (Figure 6). This OTU was in relatively high abundance in the dataset (15.5% of total reads). A histogram of read counts from this single OTU showed its abundance was positively correlated with diet amount. It was much less abundant in individuals from the lowest “R1” diet compared to higher diets (R2 and R4, Figure 6). This was consistent across all sample types and replicates, with the exception of one replicate in the blood fed adult treatment (Figure 6).

**FIGURE 7:**
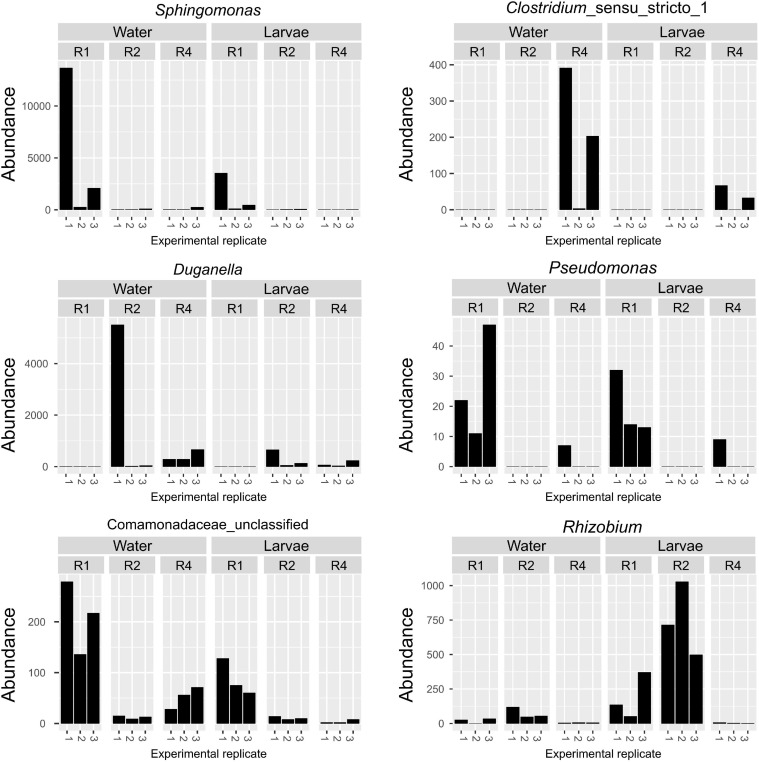
Specific OTUs are significantly affected by larval diet amount in breeding water and larvae only. Abundance of six OTUs was significantly affected by diet amounts in breeding water and larval samples. For each OTU, abundance is plotted for each sample type, diet treatment and biological replicate.

Given that the microbiota composition of adults differed substantially from that of breeding water and larvae (Figure 3), and given that the effect of diet on microbiota composition was primarily restricted to water and larval samples, we performed a subsequent ANCOM analysis on only breeding water and larval samples. Among breeding water and larval samples, we found six OTUs that differed significantly by diet (Figure 7). These OTUs mapped to the genera *Sphingomonas*, *Clostridium*, *Duganella*, *Pseudomonas*, and *Rhizobium*, and one could not be mapped below the family level *Comamonadaceae*. OTUs mapping to *Sphingomonas*, *Pseudomonas*, and *Comamonadaceae* were more abundant in R1, the lowest diet treatment compared to R2 and R4. The OTU mapping to *Clostridium* was much less abundant in R1 and R2 compared to R4, and that mapping to *Duganella* was absent in R1 but present in R2 and R4, albeit at low levels (Figure 7). Finally, the OTU mapping to *Rhizobium* was more abundant in R1 and R2 than in R4 where it was nearly absent.

## Discussion

In this work, we investigated how diet abundance in the larval breeding water affects bacterial community size and composition over the life history of *Aedes aegypti.* We found that the total amount of bacteria was lowest in samples from the lowest diet treatment (R1) and generally increased with higher larval diet abundance. We also found that diversity within samples increased with increasing diet abundance and that diet was a significant predictor of composition differences between samples. These effects were primarily observed in breeding water and larvae, however, we observed diet-induced changes in *Enterobacteriaceae* abundance that persisted into adulthood and even after blood feeding. Taken together, these findings suggest that the amount of diet available in the breeding water can influence the number of bacteria in the larval and adult midgut microbiota and these effects last long after adult eclosion. The data also suggest that nutrient availability can shift the composition of the microbiota during larval development, and that for certain OTUs, changes in composition persist into adulthood, when female mosquitoes transmit human pathogens.

The total amount of bacteria in our samples was, in general, positively correlated with larval diet abundance. This is not unexpected, as access to more nutrients in the breeding water would logically promote propagation of bacteria within the aquatic microbial community. Another non-mutually exclusive hypothesis is that shifts in the composition of the breeding water at higher larval diet abundance favor bacteria that grow to naturally higher numbers in the laboratory breeding environment or in the mosquito.

Bacterial load was lowest in R1 larvae and increased in R2 and R3 larvae. Bacterial load in R4 larvae was similar to R1 larvae despite this regimen having the highest bacterial load in the breeding water samples. Bacterial load in the larval digestive tract is logically expected to be directly related to the number of bacteria ingested. Larvae feed by sweeping water and detritus (and any accompanying bacteria) into their mouths using their brushes (Christophers, 1960), so it is probable that more bacteria in the breeding water would result in larvae ingesting more bacteria, and thus a larger microbial community in the gut. This could explain the differences we see between R1, R2, and R3, but not R4 larvae. One technical explanation for why bacterial load was low in R4 larvae is that the number of live bacteria in the gut is dynamic during development; live bacteria increase after a molt until larvae reach a critical size, at which point bacteria in the gut start to die (Coon et al., 2017). We sampled larvae at day 5 post-hatching when we anticipated they would all be 4th instars but none would have pupated. It is possible that R4 larvae were hours more advanced in their developmental timing and that the decrease in bacterial load is a result of their being closer to pupation than the other treatments. Our sampling approach was not designed to capture fluctuations in bacterial load at such hour-level resolution, and the time scale of bacterial load fluctuation in developing mosquito larvae should be considered in future investigations of diet and mosquito microbiome formation.

From a biological perspective, there are many potential hypotheses for why we observed such low numbers of bacteria in R4 despite high bacterial load in the breeding water. For example, larvae show different rates of bacterial ingestion depending on the microbes in their breeding water (Souza et al., 2019). Bacterial composition significantly shifts with increased diet, and it is therefore possible that R4 larvae experience a behavioral shift toward reduced ingestion. However, additional work is necessary to test whether the impact of diet on microbial community affects larval feeding behavior. Bacterial load could also be influenced by the ability of different bacteria to persist in the mosquito, as well as immune system signaling or other physiological processes in the mosquito that influence bacterial survival, such as pH regulation or digestion. pH in the larval digestive tract has been well documented as highly alkaline (as high as pH 11 in parts of the midgut) (Dadd, 1975; Boudko et al., 2001; Corena et al., 2002). High pH has been shown to negatively impact survival of bacteria commonly found in the mosquito breeding water and adult mosquito midgut (Lindh et al., 2008; Coon et al., 2017). It is therefore possible that shifts in composition in R4 breeding water may have favored bacteria more sensitive to alkalinity, resulting in lower overall bacterial loads. pH tolerance varies substantially even within a given bacterial genus (Barberán et al., 2017), therefore, species-level resolution would be required to determine if microbial alkalinity tolerance explains differences in bacterial load across diet treatments. Studies from other insect systems have shown that diet can influence systemic immune system signaling and susceptibility to infection in immature developmental stages, suggesting widespread connections between diet and the larval immune system (Ponton et al., 2013). It is possible that effects of diet on bacterial load in R4 larvae may be a result of differences in immune system activity. More research is needed to understand how different bacteria persist or are expelled from the larval mosquito and how mosquito larvae regulate immune signaling in response to diet and bacterial load in their environment.

Among adults, those reared with the lowest larval diet concentration had the lowest bacterial loads while those from all other treatments were significantly higher. This was consistent regardless of whether adults were sugar fed or blood fed, though bacterial loads were significantly higher overall among blood fed adults. Mosquitoes lose the vast majority of their enteric bacteria during eclosion from pupa to adult (Moll et al., 2001). Bacteria can be transstadially transmitted, though it is not clear if this happens internally or if the adult imbibes breeding water shortly after eclosion, thereby re-populating the gut with environmental bacteria (Lindh et al., 2008; Coon et al., 2014). In either case, we expect the number of bacteria in the adult midgut after eclosion to be quite low regardless of diet treatment. Differences in bacterial load after that point could potentially be influenced by ingestion of bacteria, and/or host-microbe or microbe-microbe interactions within the adult. Multiple studies have identified mechanisms that regulate the bacterial population in the adult mosquito midgut; some are driven by the bacteria themselves and some by the mosquito. For example, bacterial colonization is in some cases determined by ability of bacteria to form biofilms in the mosquito digestive tract (Hegde et al., 2019). Additionally, network analysis has shown that the presence of certain bacteria in the mosquito are significantly correlated with the presence or absence of other bacterial taxa, and the presence of certain bacteria in the digestive tract can act to reduce the numbers of other bacterial taxa (Hegde et al., 2018). This suggests that microbe-microbe interactions may play a substantial role in determining formation of the mosquito microbiota. The adult mosquito regulates the bacterial load in its gut in multiple ways, including immune system signaling (Meister et al., 2009; Clayton et al., 2013), regulating reactive oxygen species (Oliveira et al., 2011), and amino acid metabolic signaling (Short et al., 2017). Another important determinant of bacterial load is whether the mosquito is sugar fed or blood fed, as blood feeding causes rapid bacterial proliferation (Kumar et al., 2010; Oliveira et al., 2011). Low access to larval diet has been shown to affect transcript abundance of amino acid metabolism genes and many immune system genes (Telang et al., 2012; Price et al., 2015), and has also been shown to reduce melanization capacity and hemocyte number in adult mosquitoes (Suwanchaichinda and Paskewitz, 1998; Telang et al., 2012). These phenotypes were measured in whole adults, not the midgut, but they suggest multiple physiological changes that occur in response to low diet abundance that could impact bacterial load. Given our finding that low diet abundance resulted in reduced bacterial load in adult mosquitoes, further investigation is warranted to determine whether any of the potential mechanisms discussed above shape this relationship.

In addition to changes in total bacterial load, we found that diet amount significantly affected microbiota composition and diversity. As expected, alpha diversity was highest in breeding water and larvae and decreased dramatically in adults. Diet significantly affected species richness only in breeding water and higher diet correlated with significantly higher Simson’s index values across all sample types. Since richness was not affected by diet in most sample types, we can reasonably infer that increased diet primarily affected evenness, and that higher larval diet resulted in a more even distribution of sequence reads between taxa. In investigating beta diversity, we found the strongest predictor of differences between samples to be sample type, with breeding water and larvae clustering together and away from all adult samples. This is consistent with other studies, which have also documented differences in microbiota composition between breeding water, larvae, and adults (Wang et al., 2011; Gimonneau et al., 2014). Most relevant to our study, we also found that diet was a significant predictor of differences between samples, especially among breeding water and larvae. Among these sample types, we observed clustering by diet, suggesting that access to different diet levels during larval development causes significant shifts in microbiota composition.

In light of the shifts in overall diversity, we found that specific taxa were especially affected by diet, in particular *Enterobacteriaceae*. As diet amount increased, so did relative abundance of *Enterobacteriaceae*, and this difference persisted into adulthood. Linenberg et al. (2016) demonstrated that rearing *Anopheles gambiae* larvae with fish food flakes vs. fish food pellets resulted in a decrease in total amounts of *Enterobacteriaceae* and an increase in relative levels of *Flavobacteriaceae* in larvae. As adults, those reared on pellets had less *Enterobacteriaceae* than those reared on flakes (Linenberg et al., 2016). Our findings, coupled with theirs, suggest that larval diet is a significant determinant of the degree to which *Enterobacteriaceae* colonize mosquitoes across diverse genera. Bacteria from the family *Enterobacteriaceae* are commonly found in the digestive tract of mosquitoes, including those from the genera *Serratia*, *Pantoea*, *Klebsiella*, and *Enterobacter* (Guégan et al., 2018), and bacteria from this family have been tied to a number of interesting phenotypes relevant to vector borne disease transmission. For example, *Serratia* has been shown to influence susceptibility to dengue and chikungunya viruses in *Aedes aegypti* (Apte-Deshpande et al., 2012, 2014; Wu et al., 2019), susceptibility to *Plasmodium* infection in *Anopheles* (Bahia et al., 2014; Gendrin et al., 2015), and mosquito longevity (Bahia et al., 2014). Additionally, *Enterobacter* in *Anopheles* has been shown to be predictive of *Plasmodium* infection in the field and to have a significant impact on *Plasmodium* susceptibility in the laboratory (Cirimotich et al., 2011; Boissière et al., 2012). In our study, the increase in *Enterobacteriaceae* was primarily due to one OTU that mapped to the genus *Cedecea*. This OTU was at very low abundance in mosquitoes from the lowest diet (R1) but increased significantly in higher diet treatments (R2 and R4). Abundance was similar between R2 and R4, suggesting diet amount above a particular threshold does not cause additional *Cedecea* propagation. *Cedecea* has been shown to form biofilms in the digestive tract of adult mosquitoes, and biofilm formation is critical to colonization in adults (Hegde et al., 2019). It is possible that larval diet could impact abundance of these bacteria by influencing their ability to form biofilms, and future studies into this topic and other environmental drivers of bacterial colonization of mosquito midguts are warranted.

In addition to *Cedecea*, which was affected by diet across all sample types, we also identified multiple OTUs that were significantly affected by diet in only breeding water and larval samples. These included taxa identified as *Sphingomonas*, *Pseudomonas*, and an unidentified member of *Comamonadaceae*, which were all higher abundance in R1 individuals compared to R2 and R4. Interestingly, a study by Duguma et al. (2017) testing the effect of high vs. low organic matter (rabbit feed) in larval breeding water on microbiota composition in *Culex nigripalpus*, identified bacteria from the order Burkholderiales (which contains *Comamonadaceae*) as the primary indicator taxon for low organic matter. Additionally, we found that an OTU mapping to *Clostridium* was more highly abundant in R4 individuals relative to R1 or R2. In the same study, Duguma et al. found that Clostridiales (which contains *Clostridium*), was an indicator taxon for the high organic matter treatment. These findings are broadly consistent with the findings in our study. Both our study and that of Duguma et al. used rabbit chow as larval diet, however, we also supplemented our larval diet with liver powder and fish food. This suggests that enrichment of these taxa in low vs. high nutrient breeding water may be consistent across diverse genera of mosquitoes and a variety of diet types, though this requires further investigation. The consistent enrichment of these taxa in different studies of mosquito diet and microbiota formation also suggests that these relationships are reproducible under different experimental conditions (e.g., laboratory, semi-field).

Overall, we have shown that the amount of larval diet in the breeding water has a significant effect on microbiota size and composition in *Aedes aegypti*, and that these effects last into adulthood and persist after blood feeding. Nutrient levels in breeding water are variable in the field but may be predictable to some degree. For example, *A. aegypti* breed in man-made containers such as tires, but they also successfully breed in septic tanks. The nature of the nutrient content and dissolved organic material in these breeding sites is likely to be quite different and may drive predictable differences in microbiota composition. Continued investigation into the environmental drivers of variation in microbiota formation, both in controlled laboratory assays and in the field, is warranted and will provide a better understanding of how mosquito microbiomes are formed. Ultimately, these findings increase our understanding of how the microbiome may be used to understand human pathogen transmission and to develop and target interventions to reduce mosquito-borne disease transmission.

## Data Availability Statement

The data presented in the study are deposited in the NIH Sequence Read Archive, accession number PRJNA705151.

## Author Contributions

HM contributed to data collection and manuscript preparation. GD contributed to study design and manuscript preparation. SS contributed to study design, data collection, data analysis, and manuscript preparation. All authors contributed to the article and approved the submitted version.

## Conflict of Interest

The authors declare that the research was conducted in the absence of any commercial or financial relationships that could be construed as a potential conflict of interest.
