# Supplementary material for: Larval Diet Abundance Influences Size and Composition of the Midgut Microbiota of Aedes aegypti Mosquitoes
Source: Front Microbiol. 2021 Jun 18;12:645362. doi: 10.3389/fmicb.2021.645362 (PMC8249813; doi:10.3389/fmicb.2021.645362)
Supplement: Supplementary file 2 [file Table_2.docx]

| **Table S2: Primers** | | | |
| --- | --- | --- | --- |
| **Use** | **Primer name** | **Target,template** | **Sequence (5’ -> 3’)** |
| **qPCR** | **qS7-gDNA-F2** | **S7 (AAEL009496)** | **TAGACACCCTGAAGTTGTTGCAAAT** |
| **qPCR** | **qS7-gDNA-R2** | **S7 (AAEL009496)** | **TGTATATGCGCATTAGTCTCATCAA** |
| **qPCR** | **16S-F** | **16S** | **TCCTACGGGAGGCAGCAGT** |
| **qPCR** | **16S-R** | **16S** | **GGACTACCAGGGTATCTAATCCTGTT** |
