# Supplementary material for: Larval Diet Abundance Influences Size and Composition of the Midgut Microbiota of Aedes aegypti Mosquitoes
Source: Front Microbiol. 2021 Jun 18;12:645362. doi: 10.3389/fmicb.2021.645362 (PMC8249813; doi:10.3389/fmicb.2021.645362)
Supplement: Supplementary file 3 [file Table_3.docx]

| **Table S3: Change in average wing size between feeding regimens** | | | |
| --- | --- | --- | --- |
|  | **R1** | **R2** | **R3** |
| **R2** | **5.3%** |  |  |
| **R3** | **8.9%** | **3.4%** |  |
| **R4** | **8.2%** | **2.7%** | **Not significant** |
