# Supplementary material for: Larval Diet Abundance Influences Size and Composition of the Midgut Microbiota of Aedes aegypti Mosquitoes
Source: Front Microbiol. 2021 Jun 18;12:645362. doi: 10.3389/fmicb.2021.645362 (PMC8249813; doi:10.3389/fmicb.2021.645362)
Supplement: Supplementary file 4 [file Data_Sheet_1.docx]

**Models and output for all statistical analyses:**

**Wing length:**

y <- lme(winglength~diet, random=~1|replicate, data=wings)

ANOVA output:

numDF denDF F-value p-value

(Intercept) 1 86 124811.11 <.0001

Diet 3 86 46.11 <.0001

Tukey’s test output:

Linear Hypotheses:

Estimate Std. Error z value Pr(>|z|)

R2 – R1 == 0 0.14993 0.02350 6.380 < 0.001 ***

R3 - R1 == 0 0.25317 0.02402 10.542 < 0.001 ***

R4 - R1 == 0 0.23087 0.02375 9.722 < 0.001 ***

R3 - R2 == 0 0.10325 0.02377 4.344 < 0.001 ***

R4 - R2 == 0 0.08094 0.02350 3.445 0.00327 **

R4 - R3 == 0 -0.02230 0.02402 -0.929 0.78945

**qPCR Analysis:**

**Water samples**

y5 <- lme(log(CFUperuL)~diet, random=~1|exptrep/samprep, data=water.3)

ANOVA output:

|  | numDF | denDF | F-value | p-value |
| --- | --- | --- | --- | --- |
| (Intercept) | 1 | 15 | 14134.449 | <.0001 |
| diet | 3 | 15 | 5.537 | 0.0092 |

Tukey’s test output:

Linear Hypotheses:

Estimate Std. Error z value Pr(>|z|)

R2 - R1 == 0 0.3099 0.3608 0.859 0.8260

R3 - R1 == 0 0.7697 0.3608 2.133 0.1428

R4 - R1 == 0 1.3805 0.3608 3.826 <0.001 ***

R3 - R2 == 0 0.4598 0.3608 1.274 0.5793

R4 - R2 == 0 1.0706 0.3608 2.967 0.0156 *

R4 - R3 == 0 0.6108 0.3608 1.693 0.3274

**Larval samples**

y <- lme(deltact~diet, random=~1|exptrep/pool, data=larv)

ANOVA output:

numDF denDF F-value p-value

(Intercept) 1 24 2.816139 0.1063

diet 3 24 16.734185 <.0001

Tukey’s test output:

Linear Hypotheses:

Estimate Std. Error z value Pr(>|z|)

R2 - R1 == 0 -1.15183 0.20390 -5.649 < 0.001 ***

R3 - R1 == 0 -1.24772 0.20390 -6.119 < 0.001 ***

R4 - R1 == 0 -0.47449 0.20390 -2.327 0.09194 .

R3 - R2 == 0 -0.09589 0.20390 -0.470 0.96560

R4 - R2 == 0 0.67735 0.20390 3.322 0.00483 **

R4 - R3 == 0 0.77323 0.20390 3.792 < 0.001 ***

**Adult samples:**

Initial model:

y.out <- lme(deltact~larvdiet*adultdiet, random=~1|exptrep/pool, data=adult)

ANOVA output:

numDF denDF F-value p-value

(Intercept) 1 47 97.28690 <.0001

larvdiet 3 47 16.50103 <.0001

adultdiet 1 47 174.16498 <.0001

larvdiet:adultdiet 3 47 1.65180 0.1902

Final model:

y.out1 <- lme(deltact~larvdiet+adultdiet, random=~1|exptrep/pool, data=adult)

ANOVA output:

numDF denDF F-value p-value

(Intercept) 1 50 103.37848 <.0001

larvdiet 3 50 15.93362 <.0001

adultdiet 1 50 168.03627 <.0001

Tukey’s test output:

Linear Hypotheses:

Estimate Std. Error z value Pr(>|z|)

R2 - R1 == 0 -3.0163 0.5767 -5.230 <1e-04 ***

R3 - R1 == 0 -2.5035 0.5542 -4.517 <1e-04 ***

R4 - R1 == 0 -3.1498 0.5608 -5.616 <1e-04 ***

R3 - R2 == 0 0.5128 0.5326 0.963 0.770

R4 - R2 == 0 -0.1335 0.5384 -0.248 0.995

R4 - R3 == 0 -0.6463 0.5134 -1.259 0.589

Alpha diversity

**Observed species:**

Analysis of Variance Table

Response: log(alpha1$Observed)

Df Sum Sq Mean Sq F value Pr(>F)

diet.al 2 0.0584 0.0292 0.3666 0.69687

type.al 3 31.5104 10.5035 131.8460 4.817e-15 ***

diet.al:type.al 6 1.0483 0.1747 2.1931 0.07922 .

Residuals 24 1.9120 0.0797

Response: log(alpha1$Observed)

Df Sum Sq Mean Sq F value Pr(>F)

diet.al 2 0.0584 0.0292 0.296 0.7459

type.al 3 31.5104 10.5035 106.446 4.375e-16 ***

Residuals 30 2.9602 0.0987

**Chao1:**

Response: (alpha1$Chao1)

Df Sum Sq Mean Sq F value Pr(>F)

diet.al 2 53.7 26.85 1.6426 0.214493

type.al 3 7552.9 2517.64 154.0258 8.264e-16 ***

diet.al:type.al 6 377.3 62.88 3.8470 0.007889 **

Residuals 24 392.3 16.35

**Simpson index:**

Analysis of Variance Table

Response: (alpha1$Simpson)

Df Sum Sq Mean Sq F value Pr(>F)

diet.al 2 0.25729 0.12865 5.8588 0.008471 **

type.al 3 1.64258 0.54753 24.9357 1.497e-07 ***

diet.al:type.al 6 0.28642 0.04774 2.1741 0.081464 .

Residuals 24 0.52698 0.02196

Analysis of Variance Table

Response: (alpha1$Simpson)

Df Sum Sq Mean Sq F value Pr(>F)

diet.al 2 0.25729 0.12865 4.7447 0.0162 *

type.al 3 1.64258 0.54753 20.1939 2.353e-07 ***

Residuals 30 0.81341 0.02711
