# Supplementary material for: Larval Diet Abundance Influences Size and Composition of the Midgut Microbiota of Aedes aegypti Mosquitoes
Source: Front Microbiol. 2021 Jun 18;12:645362. doi: 10.3389/fmicb.2021.645362 (PMC8249813; doi:10.3389/fmicb.2021.645362)
Supplement: Supplementary file 6 [file Data_Sheet_3.docx]

#' ---

#' title: "Impact of Larval Nutrition on Mosquito Midgut Microbiome Composition Analysis"

#' author: "Sarah M. Short, The Ohio State University"

#' output:

#' pdf_document: default

#' html_document:

#' df_print: paged

#' word_document: default

#' html_notebook:

#' fig_width: 10

#' ---

#'

#' ##Set up working environment

#' Verify that working directory is set to source file location via drop down menu, and import necessary libraries:

## ------------------------------------------------------------------------

library(phyloseq)

library(ggplot2)

library(vegan)

library(biomformat)

library(nlme)

library(wesanderson)

library(RColorBrewer)

library(dplyr)

library(gridExtra)

#'

#' Note: for ordinate funciton to work in vegan for PcoA, need to install phyloseq from source:

#' install.packages("phyloseq", type="source")

#'

#'

#' ##Import data

#' Data is currently formatted as biom file. Biom file was generated in Mothur. Change taxa names to the correct nomenclature (default is numbers)

## ------------------------------------------------------------------------

larv <-import_biom("SMSSeq1amplicon16S.pick.pick.opti_mcc.0.03.biom")

larv

colnames(tax_table(larv))<- c("Kingdom", "Phylum", "Class", "Order", "Family", "Genus")

rank_names(larv)

#'

#' #Filter dataset, perform quality control steps, and assess controls:

#' Check composition of community standard and buffer controls:

## ------------------------------------------------------------------------

larv.check <- transform_sample_counts(larv, function(x) 100*x/sum(x))

larv.check

plot_bar(larv.check, x="sequencingID", fill="Phylum")

#'

#' Positive control looks mostly as expected, but with a slight bias toward proteobacteria. Should be 36% proteobacteria, 60% firmicutes.

#' Remove positive control and PCR negative control:

## ------------------------------------------------------------------------

larv.check1 <- subset_samples(larv, measurementrep!="NA")

larv.check1

#' Remove Eukaryotes, Archaea, chlorplasts, mitochondria. Cursory glance at OTUs using tax_table did not reveal any of these taxa.

## ------------------------------------------------------------------------

larv.check1 <- subset_taxa(larv.check1, (

Kingdom=="Bacteria" &

Family != "mitochondria" &

Class != "Chloroplast")

)

larv.check1

#'

#' One OTU filtered out by this step (OTU0187, appears to be mitochondria after cross-referencing with .taxonomy file)

#'

#' Remove all OTUs that contain zero reads and all taxa that are less than 0.005% of all reads in the entire dataset (Bokulich et al, 2013 https://www.ncbi.nlm.nih.gov/pmc/articles/PMC3531572/) :

## ------------------------------------------------------------------------

larv.check2 <- prune_taxa(taxa_sums(larv.check1) > 0, larv.check1)

larv.check2

sum.check <- taxa_sums(larv.check2)

sum(sum.check)#3090786

#remove taxa that are less than than 0.005% of all reads in the entire dataset:

larv.check3<- filter_taxa(larv.check2, function(x) 100*(sum(x)/3090786) > 0.005, prune=T)

larv.check3

#'

#' Perform ordination including buffer controls to see if they cluster away from experimental samples.

## ------------------------------------------------------------------------

larvbuff_nmds <- ordinate(

physeq = larv.check3,

method = "NMDS", k=3,

distance = "bray",

trymax = 100

)

plot_ordination(

physeq = larv.check3,

ordination = larvbuff_nmds,

axes=c(1,2),

color = "diet",

shape = "type",

title = "NMDS of All Samples including Buffer Blanks"

) +

scale_color_manual(values = class_colors <- c("#DA5724","#673770","#508578","orange", "black")

) +

geom_point(aes(color = diet), alpha = 0.7, size = 4) +

geom_point(colour = "grey90", size = 1.5) +

theme_bw()

plot_ordination(

physeq = larv.check3,

ordination = larvbuff_nmds,

axes=c(2,3),

color = "diet",

shape = "type",

title = "NMDS of All Samples including Buffer Blanks"

) +

scale_color_manual(values = class_colors <- c("#DA5724","#673770","#508578","orange", "black")

) +

geom_point(aes(color = diet), alpha = 0.7, size = 4) +

geom_point(colour = "grey90", size = 1.5)+

theme_bw()

#' Blanks clearly cluster separately from samples, though there is some potential overlap with R1 (low) sugar fed samples.

#' How many reads are in the buffer blanks?

#' Plot bar plot to visualize reads in all samples:

## ------------------------------------------------------------------------

plot_bar(larv.check3, x="exptrep", fill="Class", facet_grid=~type+diet)

#'

#' Buffer controls have virtually no reads. The two lowest samples after the buffer controls have 6000 reads or so, the buffer controls appear to have a few hundred at most.

#' Assess total number of reads in buffer controls:

## ------------------------------------------------------------------------

#descriptive statistics:

print(sample_sums(larv.check3))

median(sample_sums(larv.check3))

hist(log(sample_sums(larv.check3)),breaks=15, main="Distribution of reads per sample", xlab="Ln reads per sample")

#'

#' Buffer blanks are samples 128, 129, 41, 42, 85, & 86 and they have 143, 361, 66, 154, 177, 108 reads, respectively (average=144.5). This means, on average, 144.5/43205*100=0.3% of reads are potentially contaminants. They are clearly separated from other samples in this histogram of ln(read numbers).

#'

#' Conclusions regarding contamination in buffer controls: In bar plot, buffer controls look similar to samples but have a distinct signature with some firmicutes and actinobacteria. NMDS shows clear clustering of buffer samples away from experimental samples. On average, there are more than two orders of magnitude difference between the number of reads in buffer controls and those in experimental samples and average % contamination per samples is 0.3%. Given these results, continue with analysis despite minor contamination.

#'

#' Remove buffer controls from dataset:

## ------------------------------------------------------------------------

larv.1 <- subset_samples(larv.check3, type!="buffer-ctrl")

larv.1

sums.larv.1 <- as.vector(sample_sums(larv.1))

mean(sums.larv.1)

range(sums.larv.1)

sd(sums.larv.1)

#'

#' Standardize number of sample replicates for all treatment types.

#'

#' For most samples, multiple measurement replicates were taken, i.e. two or three samples from the same diet/type/experimental replicate combination. This is pseudo replication and was meant to be a way to assess repeatability of sequencing as well as prevent loss of data if one sample failed.

#' First, test whether there are significant differences between measurement replicates (i.e. technical reps collected for each diet/type/experimental rep combo)

#' To do this, perform PERMANOVA on Bray-Curtis dissimilarity values to test whether technical replicate is significant predictor:

## ------------------------------------------------------------------------

larv_checkbray <- phyloseq::distance(larv.1, method="bray")

larv.sampledf <- data.frame(sample_data(larv.1))

sample_data(larv.1)$measurementrep=as.factor(sample_data(larv.1)$measurementrep)

sample_data(larv.1)$tray=as.factor(sample_data(larv.1)$tray)

sample_data(larv.1)$exptrep=as.factor(sample_data(larv.1)$exptrep)

adonis(larv_checkbray ~ diet*type+exptrep/measurementrep, data=larv.sampledf)

#' No significant effect of technical rep on beta diversity when nested in expt rep.

#'

#' Next: remove all but one measurement rep for each diet x type combination. Reason for this is that muliple treamtent combinations have different numbers of measurement replicates (due to sequencing failing or inability to collect enough samples). For this reason, combining data from different reps would mean some diet x treatment combinations would have far larger sample size (more sequences from more samples) than others. There ARE experimental replicates built in to the design (3 larval pans/cages per diet) and that replication is still included.

#'

## ------------------------------------------------------------------------

larv.exp <- subset_samples(larv.1, measurementrep==1)

larv.exp

#Plot the number of reads for each experimental replicate/diet/sample type combination:

plot_bar(larv.exp, x="exptrep", fill="Phylum", facet_grid=~type+diet)

#'

#'

#'

#' Dataset now only contains one technical replicate per diet x sample type combination.

#'

#' #Descriptive analysis:

#'

#' Plot rarefaction curves for all samples

## ------------------------------------------------------------------------

rarecurve(t(otu_table(larv.exp)), label=F, col=c(1:36))

#'

#' All samples approach or achieve saturation. Range of 5 - 50 OTUs per sample.

#'

#' Plot a histogram showing the distribution of read counts in experimental samples:

## ------------------------------------------------------------------------

#distribution of read counts:

sum_df <- data.frame(sum=sample_sums(larv.exp))

ggplot(sum_df, aes(x=(sum))) +

geom_histogram(color = "black", fill = "indianred", binwidth = 2500) +

ggtitle("Distribution of read counts per sample") +

xlab("Read counts") +

theme(axis.title.y = element_blank())

#'

#' Descriptive statistics:

#'

## ------------------------------------------------------------------------

sum(sample_sums(larv.exp))#total reads in all samples

min(sample_sums(larv.exp))#minimum reads in any sample

max(sample_sums(larv.exp))#maximum reads in any sample

mean(sample_sums(larv.exp))#mean reads in all samples

#'

#' #Describe breakdown of most commonly represented OTUs

#'

#' Print taxonomy summary of most abundant OTUs:

#'

## ------------------------------------------------------------------------

summary(tax_table(larv.exp))

#'

#'

#'

#' Make table showing OTU proportion across all samples and the percents breakdown into phyla and families.

## ------------------------------------------------------------------------

dim(otu_table(larv.exp))#19 OTUs (rows) x 36 samples (columns)

#create a new vector containing the total number of reads for each OTU (summed over all samples)

otu.tab.sum <- NULL

for (i in 1:76) {

otu.tab.sum <- c(otu.tab.sum, sum(otu_table(larv.exp)[i,]))

}

#print new vector

otu.tab.sum

#Create table with the OTU number, taxonomy info for that OTU and the final column contains what % of all reads are from that OTU in the whole experiment:

otu.tot.pct <- as.data.frame(cbind(rownames(otu_table(larv.exp)), tax_table(larv.exp), otu.tab.sum/sum(otu_table(larv.exp))))

names(otu.tot.pct)=c("otu", "Kingdom", "Phylum", "Class", "Order", "Family", "Genus", "Proportion")

#print new table

otu.tot.pct

#make a dataframe listing each family and the total proportion of reads from that family.

fam.sums <- as.data.frame(tapply(as.numeric(as.character(otu.tot.pct$Proportion)), otu.tot.pct$Family, sum), rownames=T)

names(fam.sums) <- c("proportion")

#Verify proportions sum to 1

sum(fam.sums[,1]) #=1

#repeat at phylum level

phy.sums <- as.data.frame(tapply(as.numeric(as.character(otu.tot.pct$Proportion)), otu.tot.pct$Phylum, sum))

#Verify proportions sum to 1

names(phy.sums) <- c("proportion")

sum(phy.sums[,1]) #=1

#'

#'

#'

#' For a composition figure, remove all OTUs that are less than than 0.1% of all reads in the entire dataset:

## ------------------------------------------------------------------------

#Find total # of reads in current dataset

sum(sum.exp3 <- taxa_sums(larv.exp))#1590488

#remove taxa that are less than than 0.1% of all reads in the dataset:

larv.exp.fig <- filter_taxa(larv.exp, function(x) 100*(sum(x)/1590488) > 0.1, prune=T)

#print summary of current dataset:

larv.exp.fig

#print taxonomic distribution of OTUs in new dataset:

summary(tax_table(larv.exp.fig))

#'

#'

#'

#' Make figure showing OTU percent composition:

#'

## ------------------------------------------------------------------------

#convert to % abundnace:

larv.exp.figp <- transform_sample_counts(larv.exp.fig, function(x) 100*x/sum(x))

larv_family <- psmelt(larv.exp.figp)

#Sort abundance data by phylum, then by family, so as to group all families from the same phylum together:

sorted.ab <- arrange(as.data.frame(tax_table(larv.exp.figp)), Phylum, Family)

#make vector of each unique family name:

z <- unique(sorted.ab$Family)

p <- unique(sorted.ab$Phylum)

#relevel family in new dataset to be sorted by phylum, then by family:

larv_family$Family <- factor(larv_family$Family,levels=z)

#choose colors for graph:

class_colors <- c(brewer.pal(n=11, name="Spectral"), "plum3")

# Plot

ggplot(larv_family, aes(x = diet, y = Abundance, fill = Family)) +

facet_grid(exptrep~type) +

geom_bar(stat = "identity") + #"identity" means that bars will represent the #the values in the data, rather than a count of cases in each group

scale_fill_manual(values = class_colors) +

ylab("Relative Abundance (Family, only OTUs > 0.1%) \n") +

ggtitle("Mosquito Midgut Composition by Diet, \n Life Stage, and Experimental Replicate")

#'

#'

## ------------------------------------------------------------------------

library(exactRankTests)

library(nlme)

library(ggplot2)

ancom.W = function(otu_data,var_data,

adjusted,repeated,

main.var,adj.formula,

repeat.var,long,rand.formula,

multcorr,sig){

n_otu=dim(otu_data)[2]-1

otu_ids=colnames(otu_data)[-1]

if(repeated==F){

data_comp=data.frame(merge(otu_data,var_data,by="Sample.ID",all.y=T),row.names=NULL)

#data_comp=data.frame(merge(otu_data,var_data[,c("Sample.ID",main.var)],by="Sample.ID",all.y=T),row.names=NULL)

}else if(repeated==T){

data_comp=data.frame(merge(otu_data,var_data,by="Sample.ID"),row.names=NULL)

# data_comp=data.frame(merge(otu_data,var_data[,c("Sample.ID",main.var,repeat.var)],by="Sample.ID"),row.names=NULL)

}

base.formula = paste0("lr ~ ",main.var)

if(repeated==T){

repeat.formula = paste0(base.formula," | ", repeat.var)

}

if(adjusted==T){

adjusted.formula = paste0(base.formula," + ", adj.formula)

}

if( adjusted == F & repeated == F ){

fformula <- formula(base.formula)

} else if( adjusted == F & repeated == T & long == T ){

fformula <- formula(base.formula)

}else if( adjusted == F & repeated == T & long == F ){

fformula <- formula(repeat.formula)

}else if( adjusted == T & repeated == F ){

fformula <- formula(adjusted.formula)

}else if( adjusted == T & repeated == T ){

fformula <- formula(adjusted.formula)

}else{

stop("Problem with data. Dataset should contain OTU abundances, groups,

and optionally an ID for repeated measures.")

}

if( repeated==FALSE & adjusted == FALSE){

if( length(unique(data_comp[,which(colnames(data_comp)==main.var)]))==2 ){

tfun <- exactRankTests::wilcox.exact

} else{

tfun <- stats::kruskal.test

}

}else if( repeated==FALSE & adjusted == TRUE){

tfun <- stats::aov

}else if( repeated== TRUE & adjusted == FALSE & long == FALSE){

tfun <- stats::friedman.test

}else if( repeated== TRUE & adjusted == FALSE & long == TRUE){

tfun <- nlme::lme

}else if( repeated== TRUE & adjusted == TRUE){

tfun <- nlme::lme

}

logratio.mat <- matrix(NA, nrow=n_otu, ncol=n_otu)

for(ii in 1:(n_otu-1)){

for(jj in (ii+1):n_otu){

data.pair <- data_comp[,which(colnames(data_comp)%in%otu_ids[c(ii,jj)])]

lr <- log((1+as.numeric(data.pair[,1]))/(1+as.numeric(data.pair[,2])))

lr_dat <- data.frame( lr=lr, data_comp,row.names=NULL )

if(adjusted==FALSE&repeated==FALSE){ ## Wilcox, Kruskal Wallis

logratio.mat[ii,jj] <- tfun( formula=fformula, data = lr_dat)$p.value

}else if(adjusted==FALSE&repeated==TRUE&long==FALSE){ ## Friedman's

logratio.mat[ii,jj] <- tfun( formula=fformula, data = lr_dat)$p.value

}else if(adjusted==TRUE&repeated==FALSE){ ## ANOVA

model=tfun(formula=fformula, data = lr_dat,na.action=na.omit)

picker=which(gsub(" ","",row.names(summary(model)[[1]]))==main.var)

logratio.mat[ii,jj] <- summary(model)[[1]][["Pr(>F)"]][picker]

}else if(repeated==TRUE&long==TRUE){ ## GEE

model=tfun(fixed=fformula,data = lr_dat,

random = formula(rand.formula),

correlation=corAR1(),

na.action=na.omit)

picker=which(gsub(" ","",row.names(anova(model)))==main.var)

logratio.mat[ii,jj] <- anova(model)[["p-value"]][picker]

}

}

}

ind <- lower.tri(logratio.mat)

logratio.mat[ind] <- t(logratio.mat)[ind]

logratio.mat[which(is.finite(logratio.mat)==FALSE)] <- 1

mc.pval <- t(apply(logratio.mat,1,function(x){

s <- p.adjust(x, method = "BH")

return(s)

}))

a <- logratio.mat[upper.tri(logratio.mat,diag=FALSE)==TRUE]

b <- matrix(0,ncol=n_otu,nrow=n_otu)

b[upper.tri(b)==T] <- p.adjust(a, method = "BH")

diag(b) <- NA

ind.1 <- lower.tri(b)

b[ind.1] <- t(b)[ind.1]

#########################################

### Code to extract surrogate p-value

surr.pval <- apply(mc.pval,1,function(x){

s0=quantile(x[which(as.numeric(as.character(x))<sig)],0.95)

# s0=max(x[which(as.numeric(as.character(x))<alpha)])

return(s0)

})

#########################################

### Conservative

if(multcorr==1){

W <- apply(b,1,function(x){

subp <- length(which(x<sig))

})

### Moderate

} else if(multcorr==2){

W <- apply(mc.pval,1,function(x){

subp <- length(which(x<sig))

})

### No correction

} else if(multcorr==3){

W <- apply(logratio.mat,1,function(x){

subp <- length(which(x<sig))

})

}

return(W)

}

ANCOM.main = function(OTUdat,Vardat,

adjusted,repeated,

main.var,adj.formula,

repeat.var,longitudinal,

random.formula,

multcorr,sig,

prev.cut){

p.zeroes=apply(OTUdat[,-1],2,function(x){

s=length(which(x==0))/length(x)

})

zeroes.dist=data.frame(colnames(OTUdat)[-1],p.zeroes,row.names=NULL)

colnames(zeroes.dist)=c("Taxon","Proportion_zero")

zero.plot = ggplot(zeroes.dist, aes(x=Proportion_zero)) +

geom_histogram(binwidth=0.1,colour="black",fill="white") +

xlab("Proportion of zeroes") + ylab("Number of taxa") +

theme_bw()

#print(zero.plot)

OTUdat.thinned=OTUdat

OTUdat.thinned=OTUdat.thinned[,c(1,1+which(p.zeroes<prev.cut))]

otu.names=colnames(OTUdat.thinned)[-1]

W.detected <- ancom.W(OTUdat.thinned,Vardat,

adjusted,repeated,

main.var,adj.formula,

repeat.var,longitudinal,random.formula,

multcorr,sig)

W_stat <- W.detected

### Bubble plot

W_frame = data.frame(otu.names,W_stat,row.names=NULL)

W_frame = W_frame[order(-W_frame$W_stat),]

W_frame$detected_0.9=rep(FALSE,dim(W_frame)[1])

W_frame$detected_0.8=rep(FALSE,dim(W_frame)[1])

W_frame$detected_0.7=rep(FALSE,dim(W_frame)[1])

W_frame$detected_0.6=rep(FALSE,dim(W_frame)[1])

W_frame$detected_0.9[which(W_frame$W_stat>0.9*(dim(OTUdat.thinned[,-1])[2]-1))]=TRUE

W_frame$detected_0.8[which(W_frame$W_stat>0.8*(dim(OTUdat.thinned[,-1])[2]-1))]=TRUE

W_frame$detected_0.7[which(W_frame$W_stat>0.7*(dim(OTUdat.thinned[,-1])[2]-1))]=TRUE

W_frame$detected_0.6[which(W_frame$W_stat>0.6*(dim(OTUdat.thinned[,-1])[2]-1))]=TRUE

final_results=list(W_frame,zero.plot)

names(final_results)=c("W.taxa","PLot.zeroes")

return(final_results)

}

#'

#'

#' ##Analyiss of Differential OTU abundance between diet treatments using pairwise comparisons (Kruskal Wallis Test) followed by correction for multiple comparisons (Method/software: ANCOM)

#' Analysis was run with both rareified and unrarified data, gives the same results.

#' Make OTU data file with each sample in rows and OTUs in columns. 1st column must be sample ID

#' First run code from "ANCOM_updated_code.R" downloaded from https://sites.google.com/site/siddharthamandal1985/research (Mandal S, Treuren WV, White RA, Eggesb? M, Knight R & Peddada SD. (2015) Analysis of Composition of Microbiomes (ANCOM): A novel method for studying microbial composition, Microbial Ecology in Health and Diseases; 26: 27663.)

#'

## ------------------------------------------------------------------------

larv.exp

#transpose OTU table

ancom.otu<-t(otu_table(larv.exp))

dim(ancom.otu)

#add sample IDs as a column

ancom.otu <- cbind(row.names(ancom.otu), data.frame(ancom.otu, row.names=NULL))

#rename first column

colnames(ancom.otu)[1]<- "Sample.ID"

head(ancom.otu)

dim(ancom.otu)

ancom.meta <- sample_data(larv.exp)

dim(ancom.meta)

ancom.meta<- cbind(row.names(ancom.meta), data.frame(ancom.meta, row.names=NULL))

colnames(ancom.meta)[1]<- "Sample.ID"

ancom.meta$diet <- as.factor(ancom.meta$diet)

ancom.meta$type <- as.factor(ancom.meta$type)

head(ancom.meta)

dim(ancom.meta)

comparison_test=ANCOM.main(OTUdat=ancom.otu,

Vardat=ancom.meta,

adjusted=F,

repeated=F,

main.var="diet",

adj.formula=NULL,

longitudinal=F,

random.formula ="~1|exptrep",

multcorr=2,

sig=0.05,

prev.cut=0.95)

comparison_test$W.taxa

#'

#'

#' Print taxonomy information for significant result: OTU0003

## ------------------------------------------------------------------------

tax_table(larv.exp)[c("Otu0003"),]

#'

#' Plot abundance of OTU0003 across diets and sample types

#'

## ------------------------------------------------------------------------

otu0003 <- prune_taxa(taxa = "Otu0003", larv.exp)

otu0003

plot_bar(otu0003, x="exptrep", fill="Genus", facet_grid=~type+diet)+

theme(panel.grid.major = element_blank(), panel.grid.minor = element_blank(), panel.background = element_blank(), axis.line = element_line(colour = "black"))

#'

#'

#'

#' These analyses are done with non-subsampled dataset. Repeating with subsampled (rareified) dataset "larv.exp.sc" generated below gives the same results, as the method is robust to differences in read counts between samples (see communication from the developers for details (https://forum.qiime2.org/t/questions-regarding-ancom-input-and-output/2304/2))

#'

#' Water/Larval samples are much different than sugar fed/blood fed samples. Differences across diets in one group may be masked because all types were grouped together.

#' Split data by type (Water/Larvae in one group, Sugar fed/Blood fed in second group) and assess OTUs abundance variation across diets in these subsampled datasets:

#'

#' Break up data by type and repeat analysis:

#'

#' Water and larval samples only:

## ------------------------------------------------------------------------

larv.exp

#remove all sugar fed and blood fed adult samples:

lw <- subset_samples(larv.exp, type!="3-mg-sug")

lw <- subset_samples(lw, type!="4-mg-bf")

#transpose OTU table and make new object:

ancom.w <- t(otu_table(lw))

#Add sample IDs as new column

ancom.w.otu <- cbind(row.names(ancom.w), data.frame(ancom.w, row.names=NULL))

#rename first column:

colnames(ancom.w.otu)[1]<- "Sample.ID"

head(ancom.w.otu)

dim(ancom.w.otu)

ancom.w.meta <- sample_data(lw)

dim(ancom.w.meta)

ancom.w.meta<- cbind(row.names(ancom.w.meta), data.frame(ancom.w.meta, row.names=NULL))

colnames(ancom.w.meta)[1]<- "Sample.ID"

ancom.w.meta$diet <- as.factor(ancom.w.meta$diet)

ancom.w.meta$type <- as.factor(ancom.w.meta$type)

ancom.w.meta

dim(ancom.w.meta)

comparison_test=ANCOM.main(OTUdat=ancom.w.otu,

Vardat=ancom.w.meta,

adjusted=F,

repeated=F,

main.var="diet",

adj.formula=NULL,

longitudinal=F,

random.formula ="~1|exptrep",

multcorr=2,

sig=0.05,

prev.cut=0.95)

comparison_test$W.taxa

#'

#' Print taxonomy information for significant results:

#'

## ------------------------------------------------------------------------

tax_table(larv.exp)[c("Otu0005", "Otu0043", "Otu0011", "Otu0050", "Otu0024", "Otu0015"),]

#' Plot abundance of significant OTUs across diets and sample types:

## ------------------------------------------------------------------------

pltlist <- list()

for (i in 1:6) {

lw.sig <- c("Otu0005", "Otu0043", "Otu0011", "Otu0050", "Otu0024", "Otu0015")

pltname <- paste("otu", i, sep=".")

pltlist[[pltname]] <- plot_bar(prune_taxa(taxa=lw.sig[i], lw), x="exptrep", fill="Genus", facet_grid=~type+diet)

}

do.call(grid.arrange, c(pltlist))

#'

#' Comparable analysis of sugar+bloodfed samples is not possble, presumably becuase the vast majority of reads are in a single OTU. I tried subsampling the dataset by removing all taxa with fewer than 10 reads and all taxa that appear in fewer than 20% of samples (this is commonly done to help ANCOM run, as it does not run when reads are highly uneven in the dataset or when there is a preponderance of zeros) using the code below but the test would still not run properly.

#' #remove all OTUs with fewer than 10 reads and those that show up in fewer than 20% of samples:

#' sb.filt <- filter_taxa(sb, function(x) sum(x > 10) > (0.20*length(x)), TRUE))

#'

## ------------------------------------------------------------------------

larv.exp

sb <- subset_samples(larv.exp, type!="1-water")

sb <- subset_samples(sb, type!="2-larvae")

ancom.sb <- t(otu_table(sb))

ancom.sb.otu <- cbind(row.names(ancom.sb), data.frame(ancom.sb, row.names=NULL))

colnames(ancom.sb.otu)[1]<- "Sample.ID"

head(ancom.sb.otu)

dim(ancom.sb.otu)

ancom.sb.meta <- sample_data(sb)

dim(ancom.sb.meta)

ancom.sb.meta<- cbind(row.names(ancom.sb.meta), data.frame(ancom.sb.meta, row.names=NULL))

colnames(ancom.sb.meta)[1]<- "Sample.ID"

ancom.sb.meta$diet <- as.factor(ancom.sb.meta$diet)

ancom.sb.meta$type <- as.factor(ancom.sb.meta$type)

ancom.sb.meta

dim(ancom.sb.meta)

comparison_test=ANCOM.main(OTUdat=ancom.sb.otu,

Vardat=ancom.sb.meta,

adjusted=F,

repeated=F,

main.var="diet",

adj.formula=NULL,

longitudinal=F,

random.formula ="~1|exptrep",

multcorr=2,

sig=0.05,

prev.cut=0.90)

comparison_test$W.taxa

#' Test failed to run properly (W statistics all 0)

#'

#'

#'

#' #Scale reads to same total number based on smallest read count (rarify) using a method from Denef et al., 2016:

#'

#' Input function "scale_reads" from Denef et al., 2016 (https://www.frontiersin.org/articles/10.3389/fmicb.2016.00606/full). Actual code can be found here: (https://github.com/michberr/MicrobeMiseq/blob/master/R/miseqR.R) scale_reads is recommended by phyloseq over rarifying

#' This function scales reads by 1) determining proportions of each OTU for each sample, 2) multiplying by a given library size of n (in this case we are setting n to equal the sample with the minimum number of reads), 3) rounding down. This method is different than just subsampling all samples to contain the same number of reads, because it preserves the OTU proportions in the process.

#'

#'

## ------------------------------------------------------------------------

scale_reads <- function(physeq, n) {

physeq.scale <-

transform_sample_counts(physeq, function(x) {

(n * x/sum(x))

})

otu_table(physeq.scale) <- floor(otu_table(physeq.scale))

physeq.scale <- prune_taxa(taxa_sums(physeq.scale) > 0, physeq.scale)

return(physeq.scale)

}

#' We then subsample reads to even depth (lowest read count in dataset) using the "scale_reads function"

## ------------------------------------------------------------------------

larv.exp.sc <- scale_reads(larv.exp, n=min(sample_sums(larv.exp)))

#' Did it work? Looking at the distribution of read counts:

#'

## ------------------------------------------------------------------------

sum(sample_sums(larv.exp.sc))

min(sample_sums(larv.exp.sc))

max(sample_sums(larv.exp.sc))

mean(sample_sums(larv.exp.sc))

#' It worked!

#'

#' #Alpha Diversity:

#' Calculate diversity indices:

#' Some sources suggest not using rarified data for this (e.g. https://github.com/joey711/phyloseq/issues/287

#' http://joey711.github.io/phyloseq/plot_richness-examples.html) but the majority of published studies I read used rarified data. I am therefore using the sub-sampled dataset (scaled to sample with fewest reads)

#'

## ------------------------------------------------------------------------

alpha <- estimate_richness(larv.exp.sc, measures=c("Observed", "Chao1", "Simpson"))

alpha1 <- as.data.frame(cbind(sample_data(larv.exp.sc), alpha))

diet.al <- as.factor(alpha1$diet)

type.al <- as.factor(alpha1$type)

write.table(alpha1, "20200609-alphadiversity.csv")

alph.o.lm <- lm(log(alpha1$Observed)~diet.al*type.al)

plot(alph.o.lm)

anova(alph.o.lm)#interaction not significant

alph.o.lm1 <- lm(log(alpha1$Observed)~diet.al+type.al)

plot(alph.o.lm1)

anova(alph.o.lm1)

alph.chao.lm <- lm((alpha1$Chao1)~diet.al*type.al)

plot(alph.chao.lm)

anova(alph.chao.lm)#interaction highly significant

alph.simp.lm <- lm((alpha1$Simpson)~diet.al*type.al)

plot(alph.simp.lm)

anova(alph.simp.lm)#interaction not significant

alph.simp.lm1 <- lm((alpha1$Simpson)~diet.al+type.al)

plot(alph.simp.lm1)

anova(alph.simp.lm1)

#'

#' Plot diveristy indices:

## ------------------------------------------------------------------------

p1 <- plot_richness(larv.exp.sc, x = "diet", measures="Observed")+

facet_grid("type")+ylab("Observed")

p2 <- plot_richness(larv.exp.sc, x = "diet", measures="Chao1")+

facet_grid("type")+ylab("Chao1")

p3 <- plot_richness(larv.exp.sc, x = "diet", measures="Simpson")+

facet_grid("type")+ylab("Simpson")

grid.arrange(p1,p2,p3, nrow=3)

#'

#'

#'

#' ##Beta Diversity, comparing differences between samples:

#' Ordination of data

#' Perform NMDS using Bray Curtis distances and plot the ordination output.

#'

## ------------------------------------------------------------------------

larv_NMDS <- ordinate(

physeq = larv.exp.sc,

method = "NMDS", k=3,

distance = "bray"

)

plot_ordination(

physeq = larv.exp.sc,

ordination = larv_NMDS,

axes=c(1,2),

color = "diet",

shape = "type",

title = "NMDS of Microbiome from varying nutrition regimens"

) +

scale_color_manual(values = class_colors <- c("#DA5724","#673770","#508578","orange")

) +

geom_point(aes(color = diet), alpha = 0.7, size = 7) +

geom_point(colour = "grey90", size = 1.5)

plot_ordination(

physeq = larv.exp.sc,

ordination = larv_NMDS,

axes=c(1,3),

color = "diet",

shape = "type",

title = "NMDS of Microbiome from varying nutrition regimens"

) +

scale_color_manual(values = class_colors <- c("#DA5724","#673770","#508578","orange")

) +

geom_point(aes(color = diet), alpha = 0.7, size = 7) +

geom_point(colour = "grey90", size = 1.5)

#' PERMANOVA to test for signifiance of experimental factors "larval diet" and "sample type"

## ------------------------------------------------------------------------

larv_bray <- phyloseq::distance(larv.exp.sc, method="bray")

larv.sampledf <- data.frame(sample_data(larv.exp.sc))

adonis(larv_bray ~ exptrep+type*diet, data=larv.sampledf)

#'

#' Remove nonsignificant interaction

## ------------------------------------------------------------------------

adonis(larv_bray ~ exptrep+type+diet, data=larv.sampledf)

#'

#'

#'
