# Supplementary material for: Larval Diet Abundance Influences Size and Composition of the Midgut Microbiota of Aedes aegypti Mosquitoes
Source: Front Microbiol. 2021 Jun 18;12:645362. doi: 10.3389/fmicb.2021.645362 (PMC8249813; doi:10.3389/fmicb.2021.645362)
Supplement: Supplementary file 7 [file Image_1.pdf]

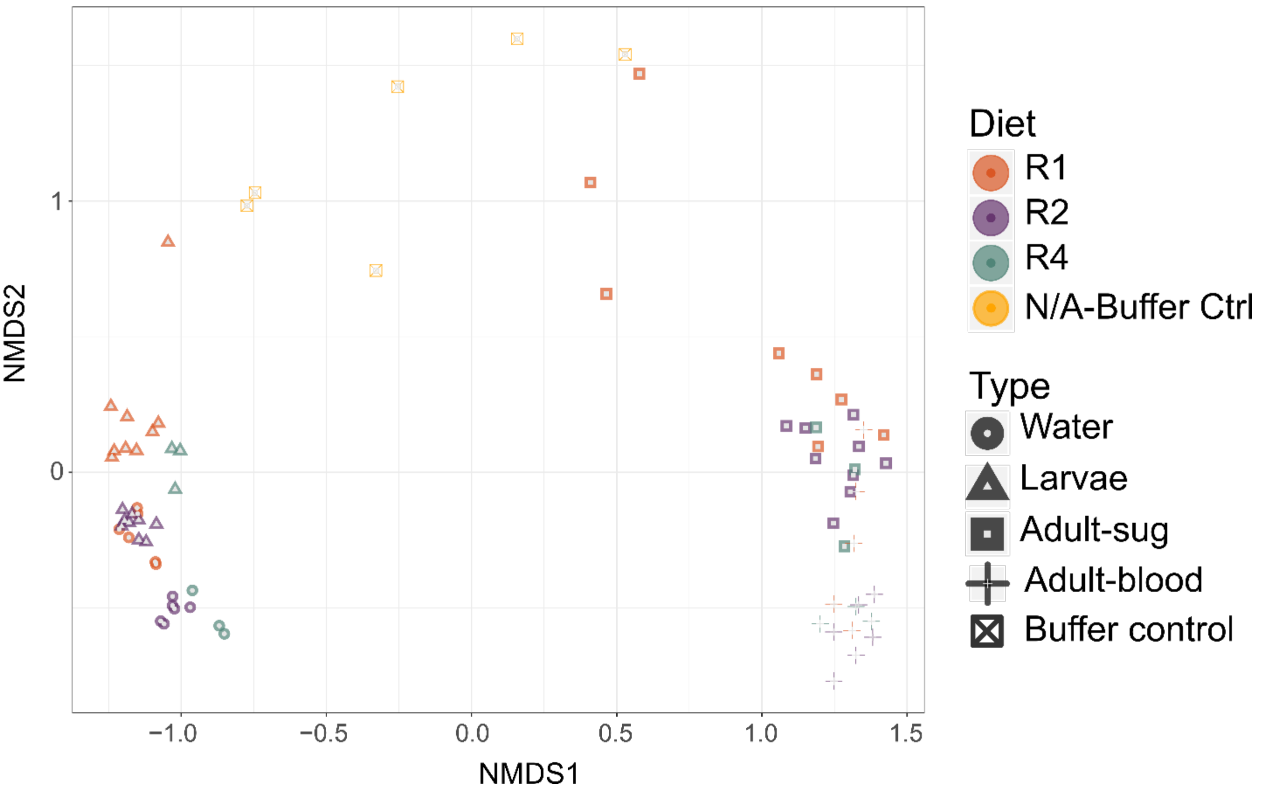

Figure S1: Non-metric multidimensional scaling analysis reveals clustering of buffer blanks separately from experimental samples. We performed an NMDS analysis using Bray-Curtis dissimilarity values for all samples, including buffer blank controls. These controls were handled identically to samples but did not contain any mosquito tissue or breeding water. NMDS revealed clustering of the blank samples (yellow squares) away from experimental samples (shaded in orange, teal, and purple).
